# Effectiveness of community-based comprehensive healthy lifestyle promotion on cardiovascular disease risk factors in a rural Vietnamese population: a quasi-experimental study

**DOI:** 10.1186/1471-2261-12-56

**Published:** 2012-07-25

**Authors:** Quang Ngoc Nguyen, Son Thai Pham, Viet Lan Nguyen, Lars Weinehall, Stig Wall, Ruth Bonita, Peter Byass

**Affiliations:** 1 Department of Cardiology, Hanoi Medical University, 1 Ton-That-Tung Street, Dong-Da District, Hanoi, 10000, Vietnam; 2 Vietnam National Heart Institute, Bach Mai Hospital, 78 Giai-Phong Avenue, Dong-Da District, Hanoi, 10000, Vietnam; 3 Umeå Centre for Global Health Research, Umeå University, Umeå, 90187, Sweden; 4 School of Population Health, Faculty of Medical and Health Sciences, University of Auckland, Auckland, 1142, New Zealand

**Keywords:** Cardiovascular disease risk factors, Healthy lifestyle promotion, Community-based intervention, Hypertension management, Quasi-experimental study, Vietnam

## Abstract

**Background:**

Health promotion is a key component for primary prevention of cardiovascular disease (CVD). This study evaluated the impact of healthy lifestyle promotion campaigns on CVD risk factors (CVDRF) in the general population in the context of a community-based programme on hypertension management.

**Methods:**

A quasi-experimental intervention study was carried out in two rural communes of Vietnam from 2006 to 2009. In the intervention commune, a hypertensive-targeted management programme integrated with a community-targeted health promotion was initiated, while no new programme, apart from conventional healthcare services, was provided in the reference commune. Health promotion campaigns focused on smoking cessation, reducing alcohol consumption, encouraging physical activity and reducing salty diets. Repeated cross-sectional surveys in local adult population aged 25 years and over were undertaken to assess changes in blood pressure (BP) and behavioural CVDRFs (smoking, alcohol consumption, physical inactivity and salty diet) in both communes before and after the 3-year intervention.

**Results:**

Overall 4,650 adults above 25 years old were surveyed, in four randomly independent samples covering both communes at baseline and after the 3-year intervention. Although physical inactivity and obesity increased over time in the intervention commune, there was a significant reduction in systolic and diastolic BP (3.3 and 4.7 mmHg in women versus 3.0 and 4.6 mmHg in men respectively) in the general population at the intervention commune. Health promotion reduced levels of salty diets but had insignificant impact on the prevalence of daily smoking or heavy alcohol consumption.

**Conclusion:**

Community-targeted healthy lifestyle promotion can significantly improve some CVDRFs in the general population in a rural area over a relatively short time span. Limited effects on a context-bound CVDRF like smoking suggested that higher intensity of intervention, a supportive environment or a gender approach are required to maximize the effectiveness and maintain the sustainability of the health intervention.

## Background

Cardiovascular diseases (CVD) account for nearly one quarter of total deaths in Vietnam and rank first among the leading causes of mortality or the cause-specific burdens of disability-adjusted life years lost [1]. Among a few established CVD modifiable risk factors, hypertension accounts for nearly 70 % of the CVD burden in the Asian-Pacific region [2] although in reality, hypertension usually clusters with other CVD risk factors [3,4]. Any strategy for comprehensive and sustainable hypertension management should always consider coexisting CVD risk factors (CVDRF) rather than only blood pressure (BP) levels [5]. Dealing with the increasing prevalence of hypertension in Vietnam [6], a community-based programme on hypertension management has been implemented since 2006 in a typical rural commune, which can be considered as a representative living environment of nearly 70 % of the Vietnamese population. In this model, there are two components integrated together: one component targets on the local hypertensive patients by monthly multidrug treatments and the other targets on the local general population by periodic healthy lifestyle promotion campaigns [7]. This paper aims to evaluate only the impacts of the latter component, the lifestyle promotion campaigns, on the progress and pattern of CVDRFs in the general population after 3 years of intervention. These findings will be important for optimizing the effectiveness of the model on management and prevention of hypertension, in order to expand it nationwide and achieve effective synergy with competing health care priorities.

## Methods

### Study setting

**Figure 1:**
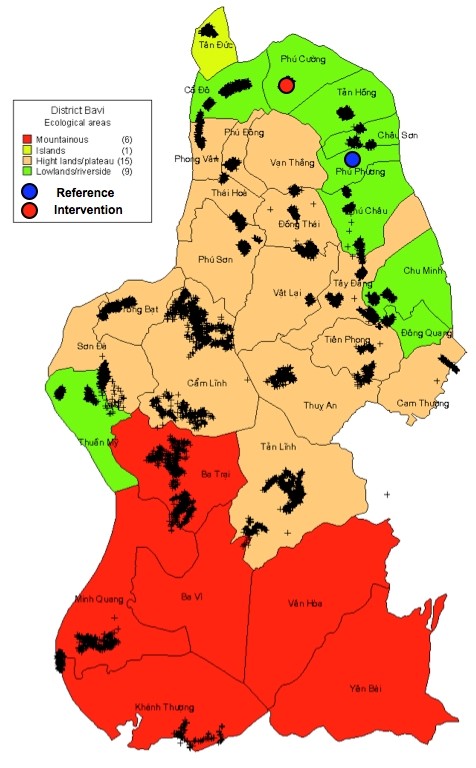
Location of intervention and reference communes in Ba-Vi District.

**Figure 2:**
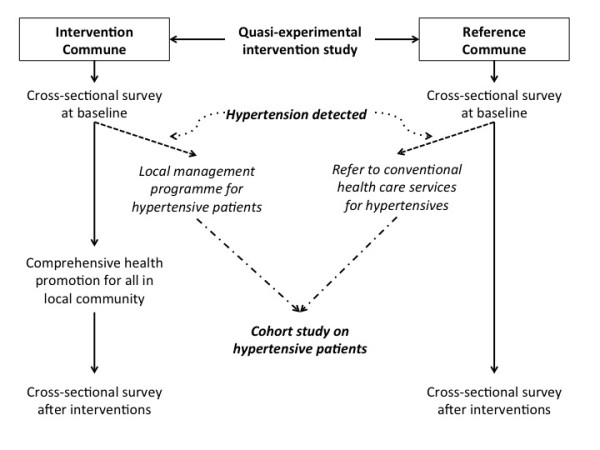
Design of the quasi-experimental trial on community-based health promotion integrated with a commune-based programme on hypertension management.

The intervention study of healthy lifestyle promotion was carried out in two typical rural communes of Ba-Vi district, 60 km to the west of Hanoi. The district had a population of about 238,000 and covered an area of 410 km^2^. Agricultural production and livestock breeding were the main economic activities of the local people (81 %) while other economic activities were forestry (8 %), fishing (1 %), small trade (3 %), handicraft (6 %) and transport (1 %). The average income per person per year in 2010 was reportedly 750 USD. Illiteracy was reportedly only 0.4 % of the adult population aged 15 years old and above. Among three representative categories of geographical areas of Ba-Vi (such as lowland, highland and mountainous, Figure 1), the category of lowland areas was chosen due to being more representative for the rural population in Vietnam. Among nine communes belong to this lowland area category, two communes were selected for a quasi-experimental study according to the following criteria: (1) average population (5,000-10,000 habitants), low rate of immigration, not including a district hospital or regional general hospital; (2) similar geographic location, population pattern, socio-economic status and local health care network; (3) low rate of drug addiction. Finally, the new hypertension management programme was implemented in Phu-Cuong commune (intervention commune) in comparison with the conventional health services pathway of cardiac care existing in Phu-Phuong commune (reference commune) (Figures 1 and 2).

### Study design

The study followed a quasi-experimental study design (Figure 2), and the impact of the intervention on the general population was evaluated by comparing randomised cross-sectional sample surveys of the general population in the two communes at baseline and after 3 years. In the intervention commune, a programme on hypertension management was implemented and integrated with the existing primary health care system, described in detail elsewhere [7]. In brief, the programme on hypertension management in the intervention commune had two integrated components simultaneously targeting two different groups: (1) one on hypertensives only, with monthly check-ups to control their blood pressure with multidrug therapy (provided at no cost), combined with lifestyle modifications and individual advice; (2) another on healthy adults in the entire commune with periodic lifestyle promotion campaigns via broadcasting, leaflets or meetings. The main messages of healthy lifestyle promotion campaigns (through mass media) focused on smoking cessation, reduction of alcohol consumption, healthier diets (reduction in salty diets and encouraging consumption of vegetables and fruits), increase in physical activity to reduce body mass index and ultimately prevent hypertension. The reference commune received only routine conventional primary health care. The choice of reference and intervention communes was made before any screening surveys or preparation activities were undertaken.

The baseline survey was conducted in December 2006 among a representative sample of 1,200 adults (≥ 25 year olds) randomly selected from whole list of local inhabitants in both intervention and reference communes, regardless of age or sex distribution. In November 2009, the evaluation cross-sectional survey, with a similar sampling strategy, was undertaken in both communes with new randomly invited candidates. A total of 1,131 and 1,189 adults from Phu-Phuong commune and 1,176 and 1,192 people from Phu-Cuong commune participated in the baseline and evaluation surveys respectively, amounting to an overall response rate of 97.7 %.

In both baseline and evaluation surveys, data were collected at local health stations in the selected communes by trained and qualified surveyors using the same questionnaire, which included personal medical history of any relevant chronic diseases, demographic background (age, sex, residential area, occupation and education level) and self-reported behavioural CVDRFs (such as smoking history, alcohol consumption, salty diet habits and level of physical activities).

### Social and cardiovascular risk factors: assessments and classification

Occupational status was classified into two groups: manual workers (farmers, building workers) and other occupations (housewives, handicraft makers, jobless, disabled). Educational level, which was determined by years of schooling and level at graduation, was classified into 2 groups: incomplete secondary schooling (≤ 9 years of education) and higher (> 9 years of education including graduation from high school or higher).

People who smoked tobacco products such as cigarettes, cigars or pipes over the previous month were classified as current smokers. People who took more than 2 standard units of drink per day (women) or more than 3 per day (men) were defined as having an excessive alcohol intake. People who self-reported preferring daily foods that contained more salt than other members in the family or people around them were classified as having salty diet. Energy requirement in metabolic equivalents (METs) for each individual was estimated based on details of duration and type of all self-reported physical activities in a typical week, following the WHO’s STEP approach. People with total physical activity less than 3,000 MET-minutes per week were classified as physically inactive [8].

In all surveys, physical measurements such as blood pressure (BP), weight, height, waist circumference (WC) and hip circumference (HC) were taken using the same standardised protocol, which has been described elsewhere [6]. BP was measured at least twice in a resting, sitting position using an automatic digital sphygmomanometer (OMRON Healthcare Inc.®, Bannockburn, Illinois), with an appropriate sized cuff. Hypertension was defined as an average systolic BP (SBP) ≥ 140 mmHg, and/or average diastolic BP (DBP) ≥ 90 mmHg, and/or self-reported current treatment with antihypertensive medications. Awareness of hypertension was defined from a self-report of any prior diagnosis of hypertension by a health care professional. Treatment of hypertension was defined as use of a prescription medication for the hypertension management at the time of the interview. Controlled hypertension was defined as pharmacological treatment of hypertension associated with an average SBP < 140 mmHg and DBP < 90 mmHg [6].

All other anthropometric measurements were performed at least twice with the participants wearing light clothing and no footwear. Body mass index (BMI) was calculated as weight (kg) divided by height squared (m^2^). Overweight was defined as BMI ≥ 23 kgm^-2^ and obesity was defined as having generalized obesity (BMI ≥ 25 kgm^-2^) or having central obesity (BMI ≥ 23 kgm^-2^ with WC either ≥ 90 cm in men or ≥ 80 cm in women), which have been specified for South Asian populations by the WHO Regional Office for the Western Pacific (WPRO) [9]. Waist–hip ratio (WHR) was defined as the ratio of the WC to the HC.

### Data analysis

The mean levels or prevalence of each CVDRF were presented as means ± standard deviation (SD) or proportions; both estimations were standardized for age and sex to compensate the difference in age and sex distribution in all surveys. Chi-square and ANOVA tests were used to identify significant differences among these prevalence or mean levels of each CVDRF between the reference and intervention communes before and after the intervention.

Considering the inter- and intra-observer variations in the same commune in different times (baseline and after 3 years), the multilevel mixed-effect linear models was used (function *xtmixed* in STATA), fitted via maximum likelihood, contained both fixed-effects and random-effects components. Using variables for survey round (0 for baseline and 1 for evaluation survey), commune (0 for reference and 1 for intervention commune), participant’s age, sex and other variables of interest for fixed-effects, it was possible to estimate the before-after changes in relevant variables after adjusting for age, sex or other explanatory variables. A survey-commune interaction term, treated as an independent variable, allowed estimation of the adjusted differences-in-differences (or impact) of relevant variables between the intervention and reference communes. The separate effectiveness of the hypertension management programme on hypertensives was adjusted in final model by adding an independent variable of being medically treated through the programme (1 for patients of the programme and 0 for the remaining regardless intervention or reference commune), and allowed to differentiate the impacts of community lifestyle promotion on the general adult population. The random-effects were random slope models adjusted for age and grouped by individual identity number (level 1) and survey-commune (level 2), to compensate for the possible inter-commune variations and the possibility that an individual could participate both in the baseline and evaluation surveys. For example, the mixed-effect model for SBP was: SBP = α + β_1_ × year + β_2_ × commune + β_3_ × (year*commune) + β_3_ × age + β_4_ × sex + β_i_ × explanatory variable _i_ + Z_1_ × commune × Z_2_ × personal identified number × (b1 × age + b_i_ × explanatory variable _i_). The value of β_1_ in model 1 is the estimation of the adjusted before-after changes in SBP in reference commune, the value of β_3_ in model 1 is the estimation of the adjusted differences-in-differences between intervention and control commune (i.e. the effectiveness of intervention), with random effect part of Z, grouped by each commune at a random slope (b_1_ and b_i_ were the intercepts for age and other explanatory variable). A significant coefficient β (i.e. its 95% CI did not cover zero) with corresponding p-value suggested the significant effect of explored variable. A p-value < 0.05 was considered to represent statistical significance.

Both descriptive and analytical statistical analyses were carried out using STATA 11 software (Stata Corporation®, Texas, USA).

### Ethical considerations

The study protocol was approved by both Scientific and Ethical Committees in Biomedical Research at Vietnam National Heart Institute-Bach Mai Hospital and Hanoi Medical University, Hanoi, Vietnam. All human subjects in the study were asked for their written consent before the collection of data, after full explanation of the goals and protocols of the study. Any participants with high blood pressure detected during the baseline survey in reference commune were referred to appropriate health facilities for further investigation and treatment. Similar patients detected during the baseline survey in the intervention commune were invited to join the newly initiated local programme on hypertension management to get monthly check-ups and drug provision. All participants from both communes had complete rights to withdraw from the study at any time without any threat or disadvantage.

## Results

A total of 4,650 adults were recruited into the final analysis (after excluding all pregnant women or missing data), of which 2,298 were from the reference commune (1,113 in baseline and 1,185 in evaluation survey) versus 2,352 from the intervention commune (1,162 in baseline and 1,190 in evaluation survey). The average age in the study population was 48.1 years for women and 51.4 for men, rather similar between baseline and evaluation surveys. The male/female ratio in our studied population was 1/1.7 similar to the results from previous national survey on CVDRFs with the same multi-stage random sampling strategy from entire list of current inhabitants and reflected the contemporary sex ratio of the rural population as a result of out-migration by men for economic reasons.

Table 1 showed the general characteristics of all cross-sectional surveys before (in 2006) and after interventions (in 2009) in the reference versus intervention communes. In comparison between the reference and intervention population at baseline, in the intervention commune there were a higher proportion of people having high education, having manual occupations; reportedly having a salty diet and having higher average BP (DBP in women, both SBP/DBP in men) than in the reference commune, which might reflect the natural difference in CVDRF patterns between the two communes before the intervention.

**Table 1:** General characteristics of studied population (separated for women and men) at the reference and intervention communes in the baseline survey and after 3 years intervention, standardized by age

**Cardiovascular disease risk factors in women**	**Baseline Survey (2006)**			**After 3-year intervention (2009)**		
	**Reference**	**Intervention**	**p**^†^	**Reference**	**Intervention**	**p**^†^
**Social risk factors**						
Age (years)	48.5 ± 14.1	47.5 ± 14.0	0.179	47.8 ± 14.7	48.6 ± 13.6	0.237
Education: secondary school or higher (%)	68.2%	78.3%	<0.001	75.9%^§^	82.1%	0.004
Occupation: manual labour (%)	17.8%	30.6%	<0.001	25.5%^§^	32.4%	0.003
**Behavioural risk factors**						
Current daily smoking (%)	0.2%	0.7%	0.146	0.2%	0.1%	0.588
Heavy alcohol consumption (%)	0.2%	0.2%	0.958	0.0%	0.0%	-
Physical inactivity (%)	8.8%	6.8%	0.150	8.4%	12.8%^§^	0.006
Salty diet (%)	19.9%	25.5%	0.011	17.5%	18.1%^§^	0.782
**Biological risk factors**						
Systolic blood pressure (mmHg)	133.7 ± 21.5	132.1 ±21.3	0.154	125.1 ±20.7^§^	121.8 ±17.5^§^	0.001
Diastolic blood pressure (mmHg)	77.6 ± 9.7	80.0 ±11.1	<0.001	76.6 ± 10.5	75.0 ± 9.5^§^	0.002
Weight (kg)	46.3 ± 6.1	46.4 ±6.5	0.743	45.3 ± 6.5^§^	47.0 ± 6.9	<0.001
Body Mass Index (kg/m^2^)	20.1 ± 2.2	20.2 ± 2.4	0.308	19.5 ± 2.4^§^	20.2 ± 2.5	<0.001
Waist circumference (cm)	67.7 ± 6.4	67.8 ± 6.7	0.884	69.7 ± 6.8^§^	71.9 ± 7.2^§^	<0.001
Hip circumference (cm)	84.3 ± 4.8	83.9 ± 4.8	0.108	84.1 ± 4.8	85.7 ± 5.2 ^§^	<0.001
Waist-Hip-Ratio	0.80 ± 0.05	0.81 ± 0.05	0.140	0.83 ± 0.06^§^	0.84 ± 0.06^§^	0.003
Prevalence of obesity	5.4%	6.8%	0.254	8.9%^§^	14.6%^§^	0.001
Prevalence of hypertension	29.1%	30.6%	0.547	20.9%^§^	19.4%^§^	0.477
Awareness among hypertensives	21.0%	35.1%	0.001	23.6%	64.6%^§^	<0.001
Being treated among hypertensives	6.9%	1.8%	0.004	10.2%	51.4%^§^	<0.001
Well controlled among hypertensives	0.6%	0.7%	0.967	0.0%	18.0%^§^	<0.001
**Social risk factors**						
Age (years)	50.8 ± 12.3	51.4 ± 12.3	0.474	50.9 ± 12.1	52.5 ± 12.3	0.048
Education: secondary school or higher (%)	81.3%	89.2%	0.001	82.0%	89.8%	0.001
Occupation: manual labour (%)	25.7%	41.4%	<0.001	30.9%	40.3%	0.004
**Behavioural risk factors**						
Current daily smoking (%)	47.9%	47.8%	0.967	45.8%	45.5%	0.930
Heavy alcohol consumption (%)	34.4%	37.9%	0.289	23.8%^§^	27.5%^§^	0.201
Physical inactivity (%)	9.0%	10.1%	0.613	11.6%	17.5%^§^	0.015
Salty diet (%)	32.1%	44.0%	0.001	30.9%	35.8%^§^	0.122
**Biological risk factors**						
Systolic blood pressure (mmHg)	136.5 ±20.4	139.4 ±20.3	0.037	132.5 ±19.0 ^§^	133.7 ±18.6 ^§^	0.355
Diastolic blood pressure (mmHg)	80.9 ± 10.3	85.3 ± 11.0	<0.001	81.8 ± 10.6	82.2 ± 11.4 ^§^	0.603
Weight (kg)	52.9 ± 7.3	52.8 ± 8.1	0.927	52.7 ± 7.9	53.3 ± 7.5	0.236
Body Mass Index (kg/m^2^)	20.3 ± 2.4	20.5 ± 2.7	0.412	19.9 ± 2.6 ^§^	20.4 ± 2.4	0.006
Waist circumference (cm)	71.0 ± 7.5	71.5 ± 8.0	0.320	74.1 ± 8.0 ^§^	74.5 ± 7.3 ^§^	0.438
Hip circumference (cm)	85.5 ± 5.6	85.0 ± 5.3	0.192	86.3 ± 5.2	86.5 ± 5.5 ^§^	0.500
Waist-Hip-Ratio	0.83 ± 0.06	0.84 ± 0.06	0.018	0.86 ± 0.06 ^§^	0.86 ± 0.05 ^§^	0.521
Prevalence of obesity	5.2%	7.6%	0.173	6.2%	5.0%	0.440
Prevalence of hypertension	34.0%	46.7%	<0.001	31.0%	39.7%^§^	0.008
Awareness among hypertensives	24.7%	31.3%	0.176	27.4%	45.0%^§^	<0.001
Being treated among hypertensives	4.7%	3.0%	0.418	15.6%^§^	36.5%^§^	<0.001
Well controlled among hypertensives	0.0%	0.4%	0.444	1.6%	8.0%^§^	0.009

^†^: in comparison between reference and intervention commune; ^§^: significant in comparison before and after 3 year interventions.

After 3 years, more women in the reference commune had high education and manual jobs compared to the baseline, but this proportion was still lower than the proportions in the intervention commune (Table 1).

Table 2 showed the changes in CVDRF patterns after 3 years intervention in the reference and the intervention communes as well as the difference between these two changes. On comparing the behavioural CVDRF patterns before and after 3-year intervention, while the prevalence of smoking unchanged in men, the reduction of heavy alcohol consumption in men was shown in both communes (p < 0.01). While being unchanged in the reference commune, the prevalence of salty diets decreased significantly in the intervention commune, both for men and for women (p < 0.01). While the prevalence of physical inactivity did not change significantly in women at reference commune, it increased in men at reference commune and both sexes at intervention communes. On comparing the biological CVDRF pattern before intervention and after 3-year, WC and WHR increased in both communes (p < 0.01). Weight and BMI was nearly unchanged in the intervention commune while these parameters reduced in the reference commune (significantly for BMI in both sexes, p < 0.05; significantly for weight only in women, p < 0.01). SBP reduced in both communes (p < 0.01) while DBP only reduced in the intervention commune (p < 0.01) (Table 2).

**Table 2:** Age-adjusted changes (95 % confidence interval) in mean or prevalence of cardiovascular disease risk factors after 3 years in the population at reference and intervention communes, estimated by multilevel linear regression model

	**Changes in reference commune after 3 years**	**Changes in intervention commune after 3 years**	**Difference-in-Difference between two communes**
**Female**			
Systolic blood pressure (mmHg)	−8.5 (−9.9; -7.2)	−11.8 (−13.1; -10.4)	−3.3 (−5.2; -1.4)
Diastolic blood pressure (mmHg)	−0.8 (−1.6; -0.1)	−5.5 (−6.3; -4.7)	−4.7 (−5.7; -3.6)
Hypertension (%)	−7.5 (−10.6; -4.4)	−11.3 (−14.4; -8.2)	−3.8 (−8.2; 0.6)
Aware hypertension (%)	3.3 (−3.7; 10.3)	25.9 (18.8; 32.9)	22.5 (12.6; 32.5)
Treated hypertension (%)	4.1 (−1.1; 9.4)	55.8 (50.4; 61.1)	51.6 (44.1; 59.1)
Controlled blood pressure (%)	−0.4 (−3.5; 2.8)	19.0 (15.8; 22.3)	19.4 (14.8; 24.0)
Weight (kg)	−0.8 (−1.2; -0.5)	0.4 (0.1; 0.8)	1.3 (0.7; 1.8)
Body Mass Index (kg/cm^2^)	−0.6 (−0.8; -0.5)	−0.1 (−0.3; 0.03)	0.5 (0.3; 0.7)
Waist circumference (cm)	2.3 (1.9; 2.8)	3.8 (3.3; 4.2)	1.5 (0.8; 2.1)
Waist Hip Ratio (cm/cm)	0.03 (0.025; 0.034)	0.03 (0.025; 0.035)	−0.001 (−0.07; 0.01)
Obesity (%)	4.2 (1.9; 6.5)	7.3 (5.1; 9.6)	3.1 (−0.1; 6.3)
Daily smoking (%)	−0.01 (−0.5; 0.5)	−0.9 (−1.4; -0.4)	−0.9 (−1.6; -0.2)
Heavy drinking (%)	−0.4 (−0.7; -0.03)	−0.2 (−0.7; 0.3)	0.2 (−0.3; 0.7)
Salty diet (%)	−2.5 (−5.9; 1.0)	−8.9 (−12.3; -5.4)	−6.4 (−11.3; -1.5)
Physical inactivity (%)	1.2 (−1.5; 3.9)	7.3 (4.6; 10.0)	6.1 (2.3; 9.9)
**Male**			
Systolic blood pressure (mmHg)	−5.0 (−7.0; -3.0)	−8.0 (−9.9; -6.2)	−3.0 (−5.8; -0.3)
Diastolic blood pressure (mmHg)	0.4 (−0.7; 1.6)	−4.2 (−5.2; -3.1)	−4.6 (−6.2; -3.0)
Hypertension (%)	−3.1 (−8.1; 1.9)	−6.6 (−11.2; -2.0)	−3.5 (−10.3; 3.3)
Aware hypertension (%)	0.1 (−9.3; 9.5)	16.0 (8.6; 23.3)	15.9 (4.0; 27.8)
Treated hypertension (%)	9.5 (2.3; 16.7)	38.9 (33.1; 44.6)	29.4 (20.2; 38.6)
Controlled blood pressure (%)	1.4 (−2.4; 5.3)	10.0 (6.9; 13.2)	8.6 (3.6; 13.6)
Weight (kg)	−0.2 (−0.8; 0.3)	0.3 (−0.2; 0.8)	0.5 (−0.2; 1.2)
Body Mass Index (kg/cm^2^)	−0.5 (−0.7; -0.3)	−0.2 (−0.3; -0.01)	0.3 (0.03; 0.6)
Waist circumference (cm)	2.8 (2.0; 3.5)	3.1 (2.5; 3.8)	0.4 (−0.6; 1.4)
Waist Hip Ratio (cm/cm)	0.03 (0.019; 0.034)	0.02 (0.015; 0.028)	−0.01 (−0.015; 0.005)
Obesity (%)	−0.7 (−0.3; 1.8)	−1.8 (−4.2; 0.5)	−1.1 (−0.5; 2.3)
Daily smoking (%)	−2.3 (−7.1; 2.6)	10.5 (−3.4; 5.5)	3.3 (−3.2; 9.9)
Heavy drinking (%)	−11.6 (−17.1; -6.2)	−9.0 (−14.0; -4.0)	2.6 (−4.6; 10.0)
Salty diet (%)	0.2 (−5.4; 5.8)	−7.5 (−12.6; -2.3)	−7.6 (−15.2; -0.03)
Physical inactivity (%)	5.2 (1.1; 9.3)	6.4 (2.6; 10.2)	1.2 (−4.3; 6.8)

**Figure 3:**
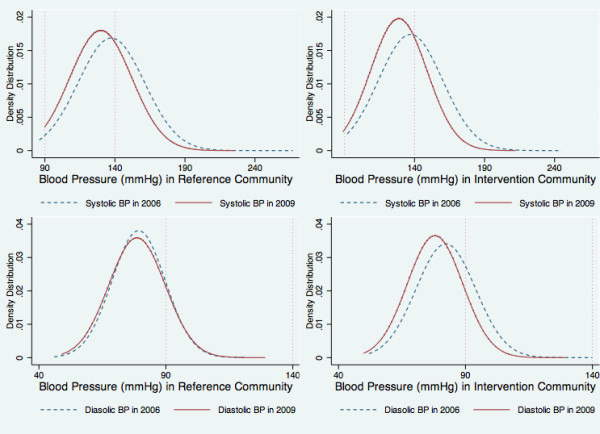
Distribution of blood pressure among 4,650 Vietnamese adults in reference commune (3a) and intervention commune (3b), at baseline (2006) and after a 3-year intervention (2009).

On comparing the changes over time between the reference and intervention communes, our estimations showed a significant increase in body size (weight, BMI and WC) and physical inactivity for women and only increase in BMI for men but a significant reduction in blood pressure (both SBP and DBP) and salty diet for both sexes after 3 years in the intervention community (Table 2). Figure 3 demonstrated the shifting to the left of the distribution curve of both SBP and DBP in the general adult population after 3-year interventions. Although the prevalence of hypertension was indifferently changes between the reference and intervention areas, the prevalence of awareness, treatment and control of hypertension significantly improved in the intervention community (p < 0.01) (Table 2).

Table 3 showed the relationship between individual BP level with other risk factors such as age, body size (BMI and WC), behavioural CVDRFs and impacts of being treated by the hypertension programme or living in intervention area, which enabled to differentiate the effects of the community-targeted component of health promotion and the patient-targeted component of hypertension management. Our estimations revealed the significant improvements of both SBP and DBP for women and DBP for men in the general adult population after intervention. In addition, this result also demonstrated that the BP changes in men related to smoking, alcohol consumption and their WC while the BP changes in women related to physical inactivity and their BMI (Table 3).

**Table 3:** Relation between individual blood pressure and other risk factors, estimated by multi level linear regression*

	**Women**		**Men**	
	**Systolic BP**	**Diastolic BP**	**Systolic BP**	**Diastolic BP**
Age (year)	0.65 (0.6; 0.7)	0.17 (0.1; 0.2)	0.57 (0.5; 0.6)	0.17 (0.1; 0.2)
Body Mass Index (kg/m^2^)	1.1 (0.7; 1.5)	0.7 (0.4; 0.9)	0.4 (−0.1; 1.0)	0.2 (−0.1; 0.5)
Waist (cm)	−0.04 (−0.2; 0.1)	0.04 (−0.1; 0.1)	0.3 (0.1; 0.4)	0.3 (0.2; 0.4)
Daily smoking (Yes vs. No)	−6.7 (−17.2; 3.7)	−0.1 (−5.8; 5.5)	−2.6 (−4.4; -0.9)	−1.5 (−2.5; -0.4)
Alcohol consumption (Heavy vs. No)	−6.0 (−25.8; 13.8)	−3.2 (−15.0; 8.6)	2.9 (1.1; 4.6)	2.2 (1.1; 3.2)
Physical Inactivity (Yes vs. No)	2.9 (0.8; 5.0)	1.3 (0.1; 2.4)	0.9 (−1.7; 3.5)	−0.7 (−2.1; 0.8)
Salty Diets (Yes vs. No)	−0.5 (−1.9; 1.0)	0.1 (−0.8; 0.9)	−1.3 (−3.0; 0.4)	−0.7 (−1.7; 0.3)
Intervention vs. Reference commune	−3.2 (−5.2; -1.1)	−5.4 (−6.6; -4.2)	−2.0 (−4.8; 1.0)	−4.4 (−6.1; -2.7)
Before vs. After 3-year intervention	−7.8 (−9.2; -6.3)	−0.5 (−1.3; 0.4)	−5.4 (−7.5; -3.2)	−0.1 (−1.4; 1.1)
Being treated in the programme on hypertension management				
- Irregular vs. No	−6.1 (−10.5; -1.7)	0.1 (−2.5; 2.6)	−2.6 (−7.9; 2.7)	−1.0 (−4.0; 2.1)
- Regular vs. No	−10.9 (−15.7; -6.0)	−4.1 (−6.8; -1.3)	−13.1 (−18.6; -7.6)	−5.4 (−8.5; -2.3)

*also adjusted for occupation, education level and marital status.

## Discussion

### Effectiveness of comprehensive health promotion interventions

After 3 years, compared to the reference, the community-targeted health intervention showed the significant reductions in SBP and DBP, and in the prevalence of salty diets, but failed to improve the prevalence of heavy alcohol consumption (reduced in both communes), smoking (unchanged) or physical inactivity (increased in the intervention commune) (Table 1).

The increased prevalence of physical inactivity and high WC suggested an increasingly sedentary lifestyle trend in our study populations, which was not uncommon in similar rural settings [10] and might reflect the epidemiological transitional status of rural populations in adopting urban lifestyles. Vietnam, like other low- and middle-income countries, is facing an emerging tide of obese and overweight individuals, which have mainly been explained by the negative effects of globalization, rapid urbanization and increasingly sedentary lifestyles [11-13]. However, our data did not show an increase in BMI, which decreased in the reference community, possibly because of diversification due to the seasonal patterns of agricultural activities.

Already identified as a major CVDRF and very common in developing countries [14], smoking prevalence was very high in Vietnamese men and probably unchanged over time [11], while it remained rare in women due to traditional and cultural resistance. Being symbolised as a metaphor for masculinity and as an indicator of modernization, men typically start smoking in teenage years, accept smoking as a culturally internalized habit, struggle against dependency [15] and are concerned about possible weight gain after smoking cessation [16]. The component of the community-targeted comprehensive health intervention in our model, which relied heavily on health education and information exchange without the back up of appropriate policies, did not impact on the prevalence of smoking in men; similar outcomes have been shown in many studies on short term or long term effects [17,18]. Failure of such gender-neutral community measures highlighted the importance of cultural and gender-specific interventions as well as the importance of a supportive smoking-free society and norms.

Our data showed the decrease of BP, especially SBP, in the reference commune even though being far less than the drop of BP in the intervention area. This decrease may be explained by reactions of local people to the baseline survey itself (the Hawthorne effect), the diffusion effects due to population mobility [19] or weight loss-induced falls in BP [20]. The two former reasons can also explain the reduction in prevalence of heavy alcohol consumption in the reference community, where there was no concurrent health intervention; nor was there any other clear socio-economic reasons. Results from multivariable analysis showed that beyond the direct effect of the hypertensive-targeted component itself on individual BP (especially for regular participants), the health promotion component still had positive impacts on DBP for both sexes and on SBP for women (Table 3). The sex-specific association between changes in BP and some behavioural CVDRFs such as physical inactivity in women and alcohol consumption in men highlighted the potentials of favourable synergistic effects from a more intensive comprehensive multi CVDRFs intervention (Table 3).

Many community intervention trials from developed countries have shown only modest or nil effects on mortality or clinical events in general populations, but substantial effects in high CVD risk patients [21], which could reflect the difficulties in assessing the true overall impact, the varying and diffuse nature of the intervention or CVDRFs themselves [22]. Similarly to limited experience from community interventions in developing countries [23-26], our study showed the short-term effect on some CVDRFs especially on BP, suggesting that lifestyle intervention can be successfully implemented in similar rural settings where the burden of hypertension is high but the awareness of them is quite low.

### Limitations of the study

Using the prevalence of CVDRFs as various markers in the study population to evaluate the effectiveness of the community health promotion intervention, our findings must be considered within the following limitations. The quasi-experimental design itself cannot guarantee comparable distributions of all properties between the two communes at baseline, so the progress of natural diversity or the diffusion of CVDRF patterns could distort the real effects of the intervention. Multi-level analysis with a random-effect model was used to minimize the data variations and take into account the dynamics of CVDRF progression. It is also possible that newly diagnosed hypertensives can easily influence neighbours, and unintentionally encourage them adopt a healthy lifestyle. Treating entire communities as study units, we used repeated cross-sectional surveys to assess the CVDRF prevalence as the outcomes. Compared to a longitudinal cohort, this approach can reduce the proportion of missing data due to outmigration or other loss to follow-up. However, it also made exploring the changes of CVDRFs over time and between communes more complex.

As with any population-based survey on CVDRFs, hypertension may have been overestimated since it was assessed only during a single visit in the survey [27], overweight status may have been underestimated depending on harvest cycles, and self-reported behavioural CVDRFs may be underestimated or have recall bias, although this should only have minimal effects on within-sample comparisons and it is an inherent problem of every epidemiological investigation. Due to the budget constraints, we could not carry out blood tests for everyone, so we might have missed any possible favourable impact of healthy lifestyle on glucose or lipid profiles.

### Future of community health promotion interventions

Using recommended top-priority population-wide interventions [28,29], our comprehensive health promotion campaigns aimed at improving the behavioural CVDRF patterns in a general population and ameliorating the adherence of hypertensives requiring lifelong treatment. Individual adherence to healthy lifestyle was associated with lower long-term risk of adverse cardiac events [30] while favourable changes in CVDRFs pattern such as smoking cessation and aggressive management of blood pressure or lipids could result in nearly 50 % decline in CVD mortality [31]. Community health promotion enabled both healthy and high CVD risk people to be exposed to the positive effects with low implementation cost (estimated total cost was only US$0.06 per capita per year in Vietnam [32]). Such a programme nationally would lead to enormous health benefits, would be pro-poor, and reduce inequalities [29], especially where the overall knowledge of CVDRFs remains relatively low [6] and in the context of budget constraints like Vietnam.

In this study, changes in CVDRF patterns, especially BP changes, were chosen as the outcome measures because they linked directly and most closely to the interventions offered, they were sensitive enough to show marginal effects or avoid potential dilution bias [19] and they can be self-witnessed, motivating local people to become involved further in CVD prevention activities. Integrating tightly with the patient-targeted component of hypertension management, our programme enabled shifts toward a strengthened primary health care system, as a part of a universal cardiac care network, to effectively and comprehensively deliver essential CVD prevention and treatment on-site and on-time by local commune-based health workers [7].

Results from our comprehensive health promotion in a rural setting showed the short-term benefits on intermediate outcomes like BP, but also demonstrated resistance to tackling behavioural CVDRFs (like smoking in men) while facing an emerging sedentary lifestyle trend, probably leading to individual clustering of CVDRFs in the general population. Despite being a matter of personal autonomy, changes in behavioural CVDRFs were often influenced and maintained over time by the objective socio-cultural environment. Any change at societal level to reduce risk exposure will be more likely to succeed than individual efforts required for self-change. Besides inevitable changes by individuals, CVDRF interventions theoretically require long-lasting social, political, and economic will to forge essential multi-level changes among communities, health systems, and health policy makers [33], such as sustained commitment and political leadership, strengthened health systems, multi-partnership cooperation, legislative frameworks, supportive environments (such as legislation to ban smoking in public or to reduce the amount of salt in processed foods, to increase taxes on alcohol, etc.), monitoring systems and accountability mechanisms [29]. In addition, the sex-specific differences on the intervention impacts in our study (e.g. the association between BP changes and other behavioural CVDRFs) suggested that a gender lens should be applied to address the gender gaps in CVDRF patterns (such as deconstructing smoking from masculine identity or specific health education for particular target groups) in order to maximize and sustain the effectiveness of the intervention in the general population.

## Conclusions

While a trend towards sedentary lifestyles emerged in the general population, the community-targeted healthy lifestyle promotion significantly improved blood BP patterns and profiles of some behavioural CVDRFs in a rural area of Vietnam, proving that lifestyle intervention is integral to a core programme on hypertension management at the local commune health station. Limited effects on a prevalent gender and context-bound CVDRF like smoking in men suggested that health interventions need higher intensities of health education, a supportive environment or a gender approach in order to optimize effectiveness and maintain sustainability in health education interventions.

## Competing interest

The authors have declared that no competing interests exist.

## Authors’ contributions

QNN designed the study, carried out the project, supervised the baseline and evaluation surveys, did the data analysis and drafted the manuscript. STP carried out the project, supervised the surveys, advised on the manuscript. VLN, SW, LW, RB, PB participated in the design of the study, advised on the implementation process and helped to write the manuscript. All authors have read and approved the final manuscript.

## Funding

The WHO in the Western Pacific Region (WHO WPRO), Vietnam Ministry of Health (MOH) and Vietnam National Heart Institute (VNHI) provided core funding support for this trial. The views expressed in this paper are those of the authors and not necessarily those of any funding body or others whose support is acknowledged. The funders had no role in study design, data collection and analysis, decision to publish, or preparation of the manuscript.

## Pre-publication history

The pre-publication history for this paper can be accessed here:

http://www.biomedcentral.com/1471-2261/12/56/prepub
